# C-756-01. Babies on the OR Board: Rethinking Pediatric Perioperative Airway Risk

**DOI:** 10.1093/jbcr/irag033.045

**Published:** 2026-04-06

**Authors:** Lauren B Nosanov, Carey K Lamphier, Allison F Linden, Ebonique Moment, Laura S Johnson

**Affiliations:** Emory University School of Medicine, Atlanta, Georgia; Grady Memorial Hospital, Atlanta, Georgia; Emory University / Children's Healthcare of Atlanta, Atlanta, Georgia; Emory University School of Medicine, Atlanta, Georgia; Grady Memorial Hospital / Emory School of Medicine / Morehouse School of Medicine, Atlanta, Georgia

## Abstract

**Introduction:**

The COVID19 pandemic served as a stark reminder of the need to consider acute respiratory issues in perioperative risk assessment. A recent process improvement (PI) evaluation of a child urgently reintubated postoperatively deemed related to undiagnosed respiratory syncytial virus prompted re-evaluation of our approach to preoperative risk stratification and operative planning for our pediatric burn patients.

**Methods:**

In 11/2024 a multidisciplinary team inclusive of burn physicians, PI nurse quality specialists, Pediatric Surgery, and Anesthesia was convened. Based on standard practice at our partner pediatric specialty hospital, the decision was made to obtain respiratory viral panel (RVP) swab testing for all admitted patients age < 16. To improve adherence the RVP panel was added to the pediatric burn admission order set in 12/2024. For RVP+ patients, interdisciplinary discussion assesses the urgency and associated risk of any proposed operative intervention. When possible, all non-emergent procedures are delayed a minimum two weeks, while elective operations including reconstruction are delayed a minimum of six weeks. Those requiring urgent intervention undergo additional preoperative discussion with Anesthesia and family counseling regarding additional risk. Extremely high-risk patients requiring urgent or emergent operations undergo timely procedures as indicated, with consideration of transfer to the pediatric facility if additional pediatric specialty services are deemed necessary.

**Results:**

Between 11/2024-6/2025, 36 pediatric patients were admitted, with 24 swabs completed on 23 patients (accounting for one readmission). Monthly testing compliance was 50% before order set integration and neared 100% after. Twelve patients yielded positive results (52%), most commonly rhinovirus/enterovirus (5) and non-COVID19 coronavirus (5). During this time, 20 acute burn operations were performed on 15 patients, four RVP+. Two children underwent operations in the first few days of their hospitalization due to injury severity, two were delayed approximately two weeks, and one underwent surgery at the children’s hospital with our team after transfer for medical management of issues not related to their burn injury. No patients experienced adverse perioperative respiratory issues.

**Conclusions:**

Adoption of a perioperative risk stratification process for pediatric burn patients involving routine admission RVP testing has been a successful intervention regarding prevention of associated morbidity. Future efforts will include monitoring long-term outcomes of patients with delayed operative intervention to assess for potential associated morbidity such as increased scarring or need for reconstruction.

**Applicability of Research to Practice:**

Introduction of routine screening practices combined with multidisciplinary risk assessment can mitigate potentially avoidable airway morbidity in pediatric burn patients.

**Funding for the Study:**

N/A.

